# Development of a universal dual-bolus injection scheme for the quantitative assessment of myocardial perfusion cardiovascular magnetic resonance

**DOI:** 10.1186/1532-429X-13-28

**Published:** 2011-05-24

**Authors:** Masaki Ishida, Andreas Schuster, Geraint Morton, Amedeo Chiribiri, Shazia Hussain, Matthias Paul, Nico Merkle, Henning Steen, Dirk Lossnitzer, Bernhard Schnackenburg, Khaled Alfakih, Sven Plein, Eike Nagel

**Affiliations:** 1 King's College London BHF Centre of Excellence, NIHR Biomedical Research Centre and Welcome Trust and EPSRC Medical Engineering Centre at Guy's and St. Thomas' NHS Foundation Trust, Division of Imaging Sciences, The Rayne Institute, London, UK; 2 Internal Medicine II, University of Ulm, Germany; 3 Internal Medicine III, University of Heidelberg, Germany; 4 Philips Healthcare, Hamburg, Germany; 5 Kings College Hospital, London, UK; 6 Academic Unit of Cardiovascular Medicine, University of Leeds, Leeds, UK

## Abstract

**Background:**

The dual-bolus protocol enables accurate quantification of myocardial blood flow (MBF) by first-pass perfusion cardiovascular magnetic resonance (CMR). However, despite the advantages and increasing demand for the dual-bolus method for accurate quantification of MBF, thus far, it has not been widely used in the field of quantitative perfusion CMR. The main reasons for this are that the setup for the dual-bolus method is complex and requires a state-of-the-art injector and there is also a lack of post processing software. As a solution to one of these problems, we have devised a universal dual-bolus injection scheme for use in a clinical setting. The purpose of this study is to show the setup and feasibility of the universal dual-bolus injection scheme.

**Methods:**

The universal dual-bolus injection scheme was tested using multiple combinations of different contrast agents, contrast agent dose, power injectors, perfusion sequences, and CMR scanners. This included 3 different contrast agents (Gd-DO3A-butrol, Gd-DTPA and Gd-DOTA), 4 different doses (0.025 mmol/kg, 0.05 mmol/kg, 0.075 mmol/kg and 0.1 mmol/kg), 2 different types of injectors (with and without "pause" function), 5 different sequences (turbo field echo (TFE), balanced TFE, k-space and time (k-t) accelerated TFE, k-t accelerated balanced TFE, turbo fast low-angle shot) and 3 different CMR scanners from 2 different manufacturers. The relation between the time width of dilute contrast agent bolus curve and cardiac output was obtained to determine the optimal predefined pause duration between dilute and neat contrast agent injection.

**Results:**

161 dual-bolus perfusion scans were performed. Three non-injector-related technical errors were observed (1.9%). No injector-related errors were observed. The dual-bolus scheme worked well in all the combinations of parameters if the optimal predefined pause was used. Linear regression analysis showed that the optimal duration for the predefined pause is 25s to separate the dilute and neat contrast agent bolus curves if 0.1 mmol/kg dose of Gd-DO3A-butrol is used.

**Conclusion:**

The universal dual-bolus injection scheme does not require sophisticated double-head power injector function and is a feasible technique to obtain reasonable arterial input function curves for absolute MBF quantification.

## Background

First-pass myocardial perfusion cardiovascular magnetic resonance (CMR) uses a series of T1-weighted images during the passage of a contrast bolus through the heart to characterize myocardial blood flow (MBF). The use of fully quantitative analysis of first-pass myocardial perfusion CMR allows the absolute quantification of MBF in units of ml/min/g and may permit an accurate, objective assessment of altered myocardial perfusion in patients with heart disease (1-3). Accurate MBF quantification by myocardial first-pass perfusion CMR relies on a linear relationship between signal intensity and gadolinium concentration. However, it is well-known that with gadolinium concentrations currently in use for first-pass perfusion MR imaging, T1-saturation effects can cause substantial signal attenuation predominantly in the left ventricular (LV) cavity where the signal intensity-time curve usually represents the arterial input function (AIF) (3, 4). To preserve an accurate AIF, previous studies using quantitative measures have focused on low doses (0.025 mmol/kg-0.05 mmol/kg) of contrast agent in combination with strongly T1 weighted sequences(1, 5). Low-dose techniques are applied for precise and reproducible absolute quantification of cardiac perfusion(1, 5). However, this approach is limited by a low contrast to noise ratio (CNR) in the myocardial tissue as a result of limited myocardial enhancement.

To overcome the limitation of T1-induced MR signal saturation in the LV blood pool and low CNR in the myocardial tissue, dual-bolus first-pass perfusion CMR methods were recently introduced to allow the use of high gadolinium concentration contrast for myocardial analysis, and a lower gadolinium concentration bolus to maintain the linearity of the LV signal intensity (6-10). These techniques use a low dose of dilute contrast agent as a prebolus before the main bolus of neat contrast agent.

Clinically important issues in the dual-bolus protocol are that:

1) Both the main-bolus of neat gadolinium contrast agent (CA), and the pre-bolus of diluted gadolinium CA solution, should be of equal volume and administered at the same flow rate(6, 7).

2) Each bolus should be followed by a saline flush to maintain a compact CA bolus in the LV chamber. Each bolus should also be equal in volume and administered at the same rate(6-9).

3) The time delay between each bolus of CA can be controlled to minimize temporal overlap, this delay can be also adjusted to heart rate if required(6-8),

4) The system should be easy to set up

5) The procedure is easy to perform and repeat within a routine clinical scan.

The dual-bolus protocol enables accurate quantification of MBF by first-pass myocardial perfusion CMR (9). However, despite the advantages and increasing demand for the dual-bolus method for accurate quantification of MBF(11), thus far, it has not been widely used in the field of quantitative perfusion CMR. The main reasons for this are that the setup for the dual-bolus method is complex and requires a state-of-the-art injector and also there is a lack of post processing software. As a solution to one of these problems, we have devised a universal dual-bolus injection scheme that does not require a sophisticated double-head power injector and can be easily employed in a clinical setting. The purpose of this study is to show the set-up and feasibility of the universal dual-bolus injection scheme.

## Methods

### Set-up for the universal dual-bolus injection scheme

The set-up for the universal dual-bolus injection scheme is described as a step-by-step protocol as follows.

**Figure 1:**
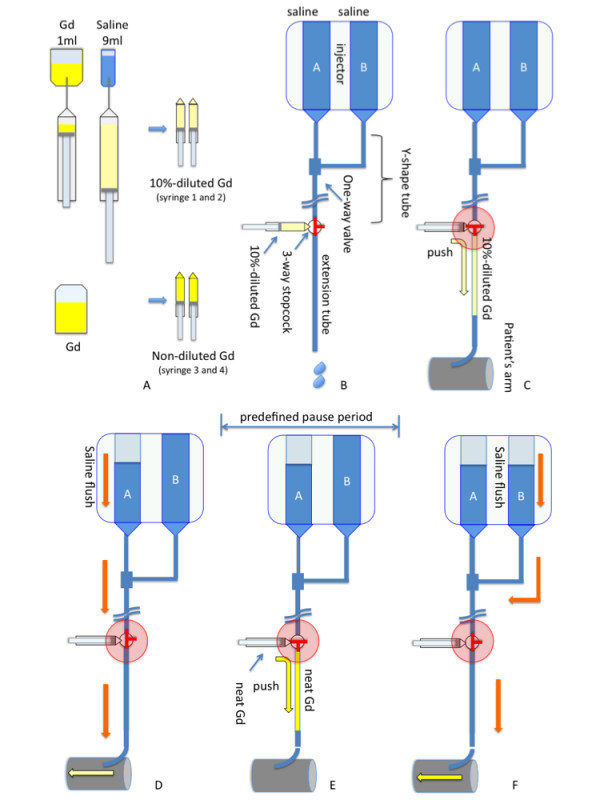
**Preparation of the dual-bolus injection scheme for injectors with a programmable "pause" functionality is illustrated**. Please see the text for details.

**Figure 2:**
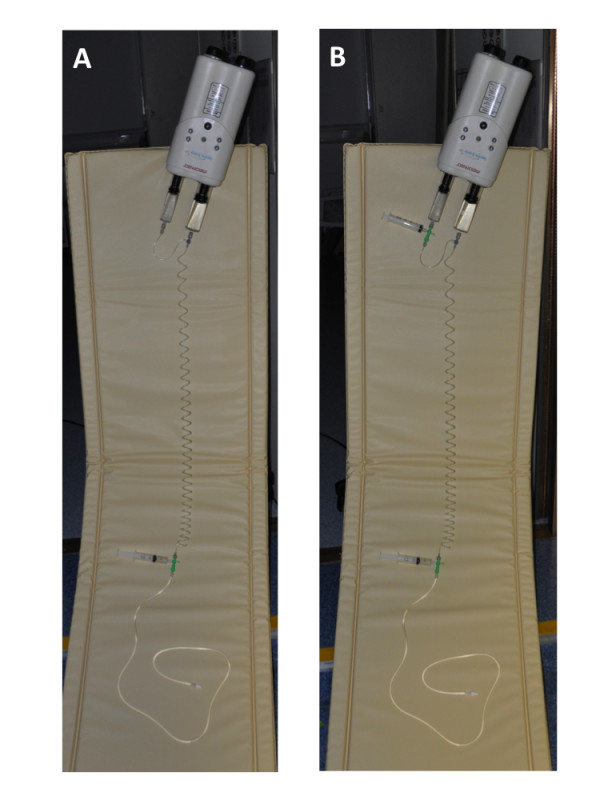
**A dual-head power injector and tubing set-up for a perfusion CMR in the case of the dual-head power injector with and without a "pause" function (A and B respectively)**. Please see the text for details.

**Figure 3:**
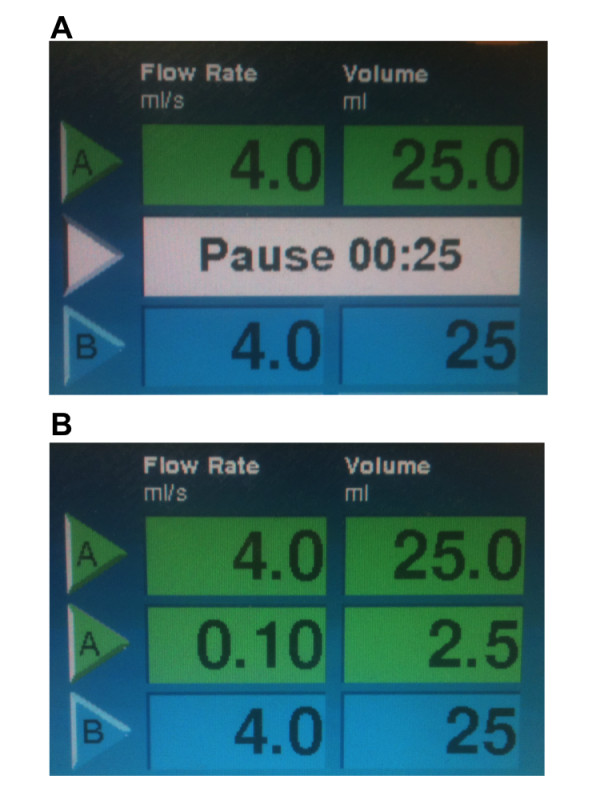
**Programmed injector control in the case of the dual-head power injector with (A) and without (B) a "pause" function**. Please see the text for details.

**For injectors with a programmable "pause" functionality **(Figure 1, 2 and 3)

Step1 (Preparation of gadolinium CA) (Figure 1A)

1. Draw 1 ml of gadolinium CA into a 10 ml syringe (syringe 1) and dilute it to a 10% solution by adding 9 ml of saline. Repeat this process for syringe 2.

2. Adjust the volume of syringe1 and syringe 2 according to the weight of the patient i.e. if a 60 kg patient needs 6 ml of 1.0 mol/L gadolinium CA then discard 4 ml of CA from 10 ml syringe (syringe 1 and 2 therefore consist of 10% dilute gadolinium CA).

3. Draw the same volume of neat gadolinium CA into two additional 10 ml syringes (syringe 3 and 4 therefore consist of neat gadolinium CA).

Note: These two pairs of syringes containing 10%-dilute and neat gadolinium CA are used for the stress and rest bolus injection respectively. It is possible to substitute larger or smaller syringes adapted to the dose of contrast medium. All syringes should be carefully labelled.

Step 2 (Preparation of injector and tubing) (Figure 1B and 2A)

1. Fill both the first (A) and second (B) power injector syringes with at least 60 ml of saline.

2. Connect Y-shaped long tube to injector syringes as per the manufacturer's instructions.

3. Connect a three-way stopcock (stopcock A) to the distal end of the Y-shaped long tube.

4. Connect a high-pressure extension tube with 15 ml volume to stopcock A.

5. Flush these tubes with saline

6. Connect the distal end of the high-pressure extension tubes to a venous cannula in the patient's antecubital vein.

Step 3 (Programming the dual-head power injector) (Figure 3A).

1. Injector A (first phase): Set the flow rate to 4 ml/s and the volume of saline flush to 25 ml).

2. Injector A (second phase): To program the delay time between the pre-bolus and the main-bolus, use the "pause" phase and set the desired time delay.

3. Injector B: Set the flow rate and volume of the saline injection as for injector A.

Step 4 (Loading the gadolinium CA and dual-bolus injection)

1. Connect the 10 ml syringe containing dilute CA (syringe 1) to stopcock A without injecting the CA (Figure 1B).

2. Once the set up for the perfusion scan is ready, arm the injector.

3. Just prior to the power injection turn stopcock A and manually inject the entire volume of dilute CA (syringe 1) into the high-pressure extension tube (Figure 1C and 1D).

4. Disconnect the syringe 1 (dilute CA) and connect the 10 ml syringe containing neat CA (syringe 3) to stopcock A without injecting the CA (Figure 1E).

5. Start the perfusion scan and the power injection at the same time.

6. During the delay time, turn stopcock A and manually inject the entire volume of neat CA from the syringe 3 (neat CA) into the high-pressure extension tube.

7. After the programmed delay, injection B (saline) starts. This allows the contrast agent within the extension tube to be pushed into the patients (Figure 1F).

**Figure 4:**
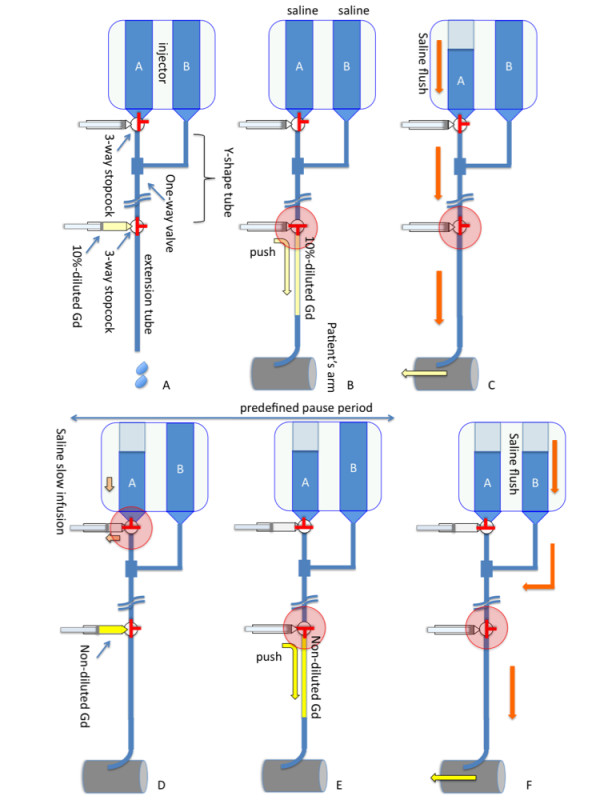
**Preparation of the dual-bolus injection scheme for injectors with no programmable "pause" functionality is illustrated**. Please see the text for details.

**For injectors with no programmable "pause" functionality **(Figure 1, 2, 3 and 4)

Step1 (Preparation of gadolinium CA) (Figure 1A)

1.-3. All steps as described above.

Step 2 (Preparation of injector and tubing)(Figure 2B and 4A)

1. Fill both the first (A) and second (B) power injector syringes with at least 60 ml of saline.

2. Connect a three-way stopcock (stopcock B) to the tip of syringe A. This enables the attachment of a syringe to act as a release mechanism for the additional saline injection, which is programmed to allow a delay between dilute and neat CA. Connect the empty 10 ml syringe to stopcock B (Figure 2B and 4A). Connect one arm of the Y-shaped long tube to stopcock B and the other arm of it to the tip of syringe B.

3. -6. These steps as described above.

Step 3 (Programming the dual-head power injector)(Figure 3B).

1. Injector A (first phase): Set the flow rate to 4 ml/s and the volume of saline flush to 25 ml.

2. Injector A (second phase): As this injector does not have a "pause" function, we need to set the flow rate and volume of the saline injection, which will allow a specific time delay. For example, for a 10s delay, 1 ml volume at 0.1 ml/sec, for a 15s delay, 3 ml volume at 0.2 ml/sec, for a 20s delay, 2 ml volume at 0.1 ml/s etc. This additional saline will then be taken up into the "release" syringe, thereby ensuring that there is no dilution of contrast agent, which will be injected into the extension tube during the delay.

3. Injector B: Set the flow rate and volume of the saline injection as for injector A.

Step 4 (Loading the gadolinium CA and dual-bolus injection)

1. -6. These steps as described above (Figure 4B).

7. Just after injection A is completed (Figure 4C), turn stopcock B to the empty 10 ml syringe in order to collect the volume injected to define the pause (step3.2). (Figure 4D).

8. -9. These steps as described above (Figure 4D).

During dynamic CMR image acquisition the patient is instructed to breath gently as the first bolus is delivered via the power injector and during the pause. The patient is subsequently instructed to hold their breath whilst the main bolus is delivered. Consequently each CA bolus, of equal volume, is delivered to the patient at the same flow rate with a pre-programmed temporal delay between the dilute and neat bolus. A second dual-bolus perfusion CMR acquisition can be performed by repeating step 3 and step 4.

#### Weight-adjusted dose of contrast agent

The volume of CA required for this method depends on which particular CA is used (0.5 mol/L or 1.0 mol/L) and on the desired dose of gadolinium CA. The dose of gadolinium contrast agent is adjusted to the patient's weight. The current injection scheme has been developed for a 0.1 mmol/kg dose of Gd-DO3A-butrol (Gadovist ^®^, Schering, Germany) (1.0 mol/L). In this setting, the volume of gadolinium CA required is less than 10 ml for an average-sized individual. We, therefore, use 10 ml small syringes and 15 ml extension tubes for the set-up described in this article. However, with minor modifications (e.g. using shorter or longer extension tubes and smaller or larger syringes), this scheme can be applied to any type and dose of commonly used commercially available gadolinium CA.

### Validation studies

Adenosine stress and/or rest dual-bolus perfusion CMR were performed mainly in patients with known or suspected coronary artery disease using the universal dual-bolus injection scheme. For perfusion CMR, three short axis slices were acquired every heart beat for a period lasting 70 heartbeats using one of the following non-slice-selective saturation-recovery perfusion sequences; turbo field echo (TFE)/turbo fast low-angle shot (TurboFLASH), balanced TFE, k-space and time (k-t) accelerated TFE and k-t accelerated balanced TFE (Table 1). In addition to these 4 different sequences, three different contrast agents (Gd-DO3A-butrol (Gadovist^®^, 1 mol/L, Schering, Germany), Gd-DTPA (Magnevist^®^, 0.5 mol/L, Schering, Germany) and Gd-DOTA (Dotarem^®^, 0.5 mol/L, Laboratoire Guerbet, France)), 4 different doses (0.025 mmol/kg, 0.05 mmol/kg, 0.075 mmol/kg, 0.1 mmol/kg), 2 different injectors (with and without "pause" function; Spectris^® ^and Spectris Solaris^® ^EP, respectively; MEDRAD, INC., USA) and 3 different MR scanners from 2 different manufacturers (Philips Achieva and Intera; Siemens Avanto) were tested (Table 2). Dilution of the gadolinium contrast agent was performed by a physician at each CMR session. The preparation time for the dual-bolus set-up and heart rate at rest and during adenosine stress was recorded. Stroke volume (SV) and ejection fraction (EF) were determined from standard short-axis cine MR images covering the entire left ventricle using a balanced steady state free precession (b-SSFP) sequence(12). Cardiac output at rest and during stress was calculated as the stroke volume multiplied by the heart rate at rest and during stress respectively. In the current study, we didn't perform a series of contiguous short axis cine MRI during adenosine stress. Instead, we used the rest left ventricular stroke volume (SV) to estimate the cardiac output (CO) during stress as; CO_stress _= SV_rest _× HR_stress_, where HR is heart rate. In this way, cardiac output during stress can be overestimated. However, in terms of the purpose of this study to validate the dual-bolus method setup, this over estimation of stress cardiac output doesn't affect the overall results of our study, because the delay time between pre-bolus and neat bolus is always less during stress than in the resting state.

**Table 1:** Scan parameters of the saturation-recovery perfusion CMR sequences

Sequences	Manufacturer	Magnetic field strength	Delay between saturation preparation pulse and center of k-space	TR (ms)	TE (ms)	**FA **(°)
TFE	Philips	3.0	105	3.6	1.7	18

k-t accelerated TFE	Philips	3.0	110	2.7	0.9	20

TFE	Philips	1.5	100	3.8	1.8	18

b-TFE	Philips	1.5	100	2.5	1.2	50

k-t accelerated b-TFE	Philips	1.5	100	2.9	1.5	50

TurboFLASH	SIEMENS	1.5	255	156	1.13	12

(TR = repetition time, TE = echo time, FA = flip angle, TFE = turbo field echo, b-TFE = balanced TFE, k-t = k-space and time, TurboFLASH = turbo fast low-angle shot)

**Table 2:** Number of dual-bolus perfusion scans (patients) in which different kinds and doses of gadolinium contrast agent, manufacturers and perfusion sequences were used

Gd-DO3A-butrol 1.0 mol/L	Philips 3.0T		
	
Dose (mmol/kg)	TFE	k-t accelerated TFE	
	
0.025	28 (14)	-	
	
0.075	-	1 (1)	
	
0.1	-	4 (2)	
Gd-DO3A-butrol 1.0 mol/L	Philips 1.5T		

Dose (mmol/kg)	TFE	b-TFE	k-t accelerated b-TFE

0.025	4 (2)	-	-

0.075	2 (2)	-	19 (11)

0.1	-	-	37 (19)

0.1	-	2 (2)	33 (18)

Gd-DO3A-butrol 1.0 mol/L	SIEMENS 1.5T		
		
Dose (mmol/kg)	TurboFLASH		
		
0.05	6 (3)		
		
0.075	3 (2)		
		
Gd-DTPA 0.5 mol/L	Philips 1.5T		
		
Dose (mmol/kg)	b-TFE		
		
0.05	10 (5)		
		
Gd-DOTA 0.5 mol/L	Philips 1.5T		
		
Dose (mmol/kg)	b-TFE		
		
0.1	12 (6)		

(TFE = turbo field echo, b-TFE = balanced TFE, k-t = k-space and time, TurboFLASH = turbo fast low-angle shot)

To test the feasibility of the dual-bolus set-up, we sent a description of this dual-bolus method (i.e. the subsection entitled "Set-up for the universal dual-bolus injection scheme" in this paper) to 3 different sites in different countries and asked them to perform perfusion scans in 5 patients using these methods and predefined pause of 25s.

### Data analysis

**Figure 5:**
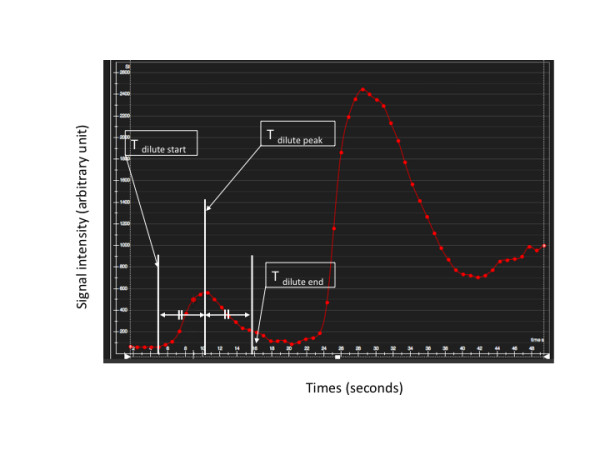
**In the time-signal intensity plot, several time frames of interest from arterial input function curve are indicated: dilute start frame (T **_**dilute start**_)**, dilute peak frame (T **_**dilute peak**_**), dilute end frame (T **_**dilute end**_**), neat start frame (T **_**neat start**_**)**. T _dilute end _was specified the time point as; (T _dilute peak_- T _dilute start_) + T _dilute peak_. The time width of dilute CA bolus curve (TW _dilute_) was defined as T _dilute end _- T _dilute start_.

Adenosine stress and rest perfusion CMR images were analyzed using dedicated software (CMR 42; Circle Cardiovascular Imaging Suite 12, Calgary, Alberta, Canada). On a representative image from the dynamic series, an observer manually placed a circular region of interest (ROI) in the LV blood pool depicted on a basal slice to obtain the time-signal intensity plot of the arterial input function. The ROI was then copied to the other dynamic images of the same slice, the positions reviewed and manually adjusted to correct for respiratory motion during data acquisition if required. In the time-signal intensity plot (Figure 5), we defined several time points of interest from the arterial input function curve namely: dilute start point (T _dilute start_), dilute peak point (T _dilute peak_) and dilute end point (T _dilute end_). T _dilute end _was specified the time point as; (T _dilute peak_- T _dilute start_) + T _dilute peak_. The time width of dilute CA bolus curve (TW _dilute_) was defined as T _dilute end _- T _dilute start _(Figure 5). If TW _dilute _was shorter than the predefined pause, this was regarded as an overlap between the AIF curves of the dilute and neat CA bolus. This would affect the quantitative analysis negatively. The relationship between TW _dilute _and cardiac output was obtained in 36 patients who underwent stress and rest dual bolus perfusion scan using 0.1 mmol/kg of Gd-DO3A-butrol (1 mol/L).

### Statistics

Linear regression analysis was performed in the 36 patients who underwent stress and rest dual bolus perfusion scan using 0.1 mmol/kg of Gd-DO3A-butrol (1 mol/L) to evaluate the correlation of cardiac output and TW _dilute _using MedCalc, version 11.4, software (MedCalc Software bvba, Mariakerke, Belgium). For all continuous parameters, results are given as the mean ± standard deviation. A p value of less than 0.05 was considered as statistically significant.

## Results

130 dual-bolus perfusion scans were performed in 70 patients at the original site where the dual-bolus scheme was devised. 31 scans in a further 16 patients were subsequently completed at 3 different sites. In total 161 dual-bolus perfusion scans were performed, 41 (25%) of these were performed using a dual-head power injector with a "pause" function. Three technical errors (1.9%) were observed in the 161 perfusion scans. Two of three errors were observed at the original site. In these two cases, the dilute contrast was confused with neat one resulting in the neat bolus being administered first. The remaining error was observed at a remote site, the most likely cause was related to manual injection of dilute contrast into the extension tube at a wrong time. No power injector related errors were observed. Apart from these 3 errors, all dual-bolus perfusion scans were successfully completed. The preparation time for the dual-bolus set-up was 6.9 ± 1.5 min.

For all patients, EF and heart rate at rest and during stress were 57 ± 15% (range 81-16%), 68 ± 13 beat/min (range 43-106 beat/min) and 90 ± 17 beat/min (range 48- 167 beat/min), respectively. Cardiac output was 5.6 ± 1.5 L/min (range 9.6-2.4 L/min) at rest and 7.4 ± 2.1 L/min (range 3.4-14.0 L/min) during stress.

**Figure 6:**
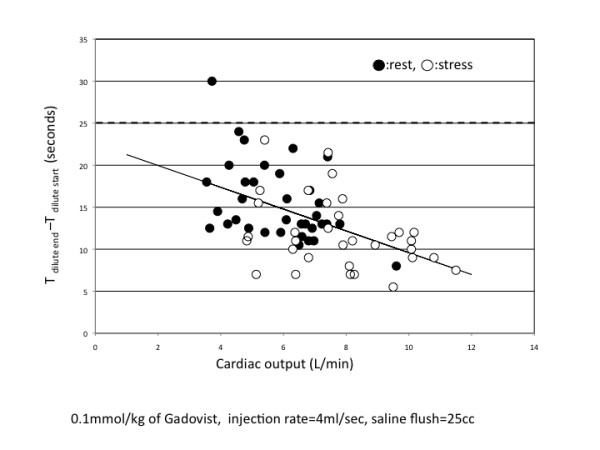
**Linear regression analysis showed a moderate correlation between TW **_**dilute **_**and cardiac output in the group A (0.1 mmol/kg of Gd-DO3A-butrol, n = 36) (y = -1.2978x + 22.559, r = 0.511, p < 0.001)**. This graph also indicated that 25s is the optimal duration for the predefined pause despite of one outlier in the patient with low cardiac output.

Linear regression analysis showed a moderate correlation between TW _dilute _and cardiac output (y = -1.2978 × + 22.559, r = 0.511, p < 0.001) (Figure 6). This plot also indicated that 25s is the optimal duration for the predefined pause despite one outlier who was a patient with low cardiac output. TW _dilute _at rest was significantly longer than TW _dilute _during stress (15.6 ± 4.6s vs 11.8 ± 4.2s, p < 0.001).

**Figure 7:**
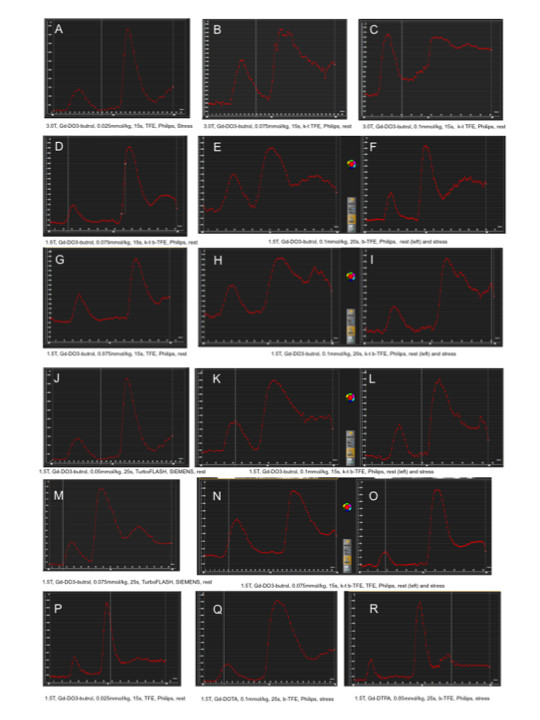
**Raw time-signal intensity curves for dual-bolus first-pass perfusion MR imaging of LV blood pool are illustrated for the following each condition:** (A: 3.0T, Gd-DO3-butrol, 0.025 mmol/kg, TFE, Philips, Stress, B: 3.0T, Gd-DO3-butrol, 0.075 mmol/kg, k-t TFE, Philips, rest, C: 3.0T, Gd-DO3-butrol, 0.1 mmol/kg, k-t TFE, Philips, rest, D: 1.5T, Gd-DO3-butrol, 0.075 mmol/kg, k-t b-TFE, Philips, rest, E: 1.5T, Gd-DO3-butrol, 0.1 mmol/kg, b-TFE, Philips, rest, F: 1.5T, Gd-DO3-butrol, 0.1 mmol/kg, b-TFE, Philips, stress, G: 1.5T, Gd-DO3-butrol, 0.075 mmol/kg, TFE, Philips, rest, H:1.5T, Gd-DO3-butrol, 0.1 mmol/kg, k-t b-TFE, Philips, rest, I: 1.5T, Gd-DO3-butrol, 0.1 mmol/kg, k-t b-TFE, Philips, stress, J: 1.5T, Gd-DO3-butrol, 0.05 mmol/kg, TurboFLASH, SIEMENS, rest, K: 1.5T, Gd-DO3-butrol, 0.1 mmol/kg, k-t b-TFE, Philips, rest, L: 1.5T, Gd-DO3-butrol, 0.1 mmol/kg, k-t b-TFE, Philips, stress, M: 1.5T, Gd-DO3-butrol, 0.075 mmol/kg, TurboFLASH, SIEMENS, rest, N: 1.5T, Gd-DO3-butrol, 0.075 mmol/kg, k-t b-TFE, TFE, Philips, rest, O: 1.5T, Gd-DO3-butrol, 0.075 mmol/kg, k-t b-TFE, TFE, Philips, stress, P: 1.5T, Gd-DO3-butrol, 0.025 mmol/kg, TFE, Philips, rest, Q: 1.5T, Gd-DOTA, 0.1 mmol/kg, b-TFE, Philips, stress, R: 1.5T, Gd-DTPA, 0.05 mmol/kg, b-TFE, Philips, stress (magnetic field strength, kind of gadolinium CA, dose of gadolinium CA, sequence, manufacturer and rest or stress, respectively)). Two peaks were cascaded to produce a continuous time-signal intensity plot: a lower peak after 10%-dilute contrast administration, followed by a higher peak after neat contrast administration. Figure 5E and 5F, 5H and 5I, 5K and 5L and 5N and 5O are the signal-intensity curves for rest and stress perfusion obtained in the same session. These graphs show that this dual-bolus approach has a good reproducibility in the same CMR session for rest and stress perfusion.

The dual-bolus scheme worked well if the appropriate predefined pause was selected for any of the following conditions: three different contrast agents (Gd-DO3A-butrol, 1 mol/L; Gd-DTPA, 0.5 mol/L; Gd-DOTA 0.5 mol/L), 4 different doses (0.025 mmol/kg, 0.05 mmol/kg, 0.075 mmol/kg, 0.1 mmol/kg), 2 different types of injectors (with and without "pause" function) and 3 different MR scanners from 2 different manufacturers (Philips Acheiva and Intera; Siemens Avanto) (Figure 7).

**Figure 8:**
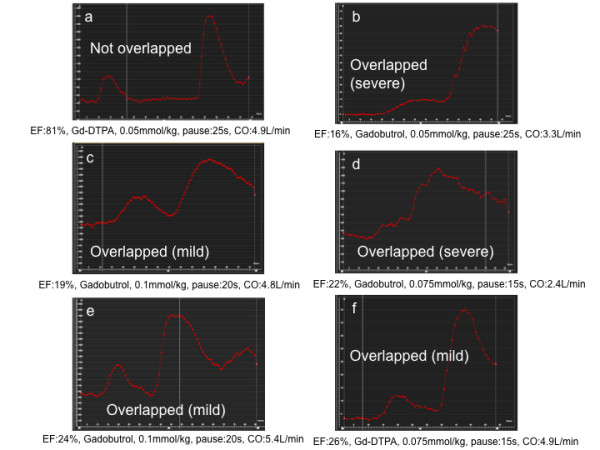
**Time-signal intensity curves for dual-bolus perfusion scan at rest in a patient with hyper contractile LV EF (>80%) (a) and with low LV EF (<30%) (b, c, d, e, f) are shown**. The information under each curve is ordered as EF, contrast agent, dose, predefined pause, CO (EF: ejection fraction, CO: cardiac output). In the patient with hyper contractile LV EF, two curves, namely, dilute and neat CA bolus, are well separated. However, in the patients with low LV EF, these two curves are overlapped with a various extent regardless of CA dose and predefined pause duration.

There was 1 patient with hyper contractile LV function (EF >80%) and 5 patients with low LV function (EF < 30%) in this study patient population (Figure 8). In all patients with an EF < 30% the time-signal intensity curves showed an overlap between the dilute and neat CA bolus curves regardless of the duration of the predefined pause. In contrast there was excellent separation of the two curves in the case with hyper contractile LV function.

## Discussion

The universal dual-bolus injection scheme proposed in the present paper has several advantages. Firstly, this method is not dependent on sophisticated function of a double-head power injector. The latest dual-head power injectors have variable functions such as a multiple injection phase, a "pause" phase, a "hold" phase and so on. In some newer injectors, it is even possible to selectively inject gadolinium CA or saline interchangeably. However, most of the power injectors widely available for clinical CMR have only two injection phases for each injector head and no "pause" or "hold" phase. The order of contrast injection and saline injection cannot be programmed in these injectors. With these injectors, if the syringes on the injector heads are set up in the usual way and filled with contrast media for the first injection and saline for the second injection, it is complex to inject the dilute contrast medium and neat gadolinium CA serially and impossible to program the temporal delay between each bolus. In the present study, we used 2 different double-head injectors with and without a "pause" phase and found no injector related errors. The dual-bolus injection scheme described in this article provides a practical, straightforward and robust solution even for standard injectors. Secondly, using this method of manually injecting the contrast agent into the tube just before the injection, the dual bolus can be easily and reliably repeated in every CMR perfusion scan. Repeatability of the dual-bolus method is important in clinical assessment of ischemic heart disease because both stress and rest perfusion CMR are done in the same session. In the current study, 2 remote and 1 local site demonstrated that this dual-bolus scheme can be easily and reliably repeated. Both sites completed successful dual-bolus perfusion scans with minimal training. Although Christian TF et al performed similar methods for their dual-bolus method in their animal study, they did not repeat the dual-bolus injection in the same session(8). Recently we have proposed an isolated perfused pig heart model for novel sequence development(13). This model is well controlled, offers exact titration of coronary blood flow and has also proven amenable for perfusion imaging using the dual bolus contrast injection scheme. The particular advantage of the pig heart model is that one can perform multiple perfusion scans in a single heart. It is considered an important vehicle for validation of various approaches for quantification of myocardial blood flow before translating those methods to patients. Ritter et al quantified stress and rest MBF by perfusion CMR in healthy volunteers with the pre-bolus technique (14). However, practically the pre-bolus technique requires a different set up because this technique uses 1 ml of undiluted contrast medium for the first bolus to determine the arterial input function. This pre-bolus technique also requires different post processing to construct the AIF from the pre-bolus time-intensity curve. There is only a single study by Utz et al. which applied a dual-bolus technique to stress and rest perfusion CMR in patients (9). However, although they demonstrated improved accuracy for absolute MBF values compared to the single-bolus approach, the details of the dual-bolus injection scheme are not provided. Thirdly, this dual-bolus injection scheme can be performed without any new, unusual or expensive materials or techniques. The only material required in addition to the normal set-up are extension tubes, 3-way stopcocks and syringes which are all widely available.

The regression analysis obtained in this study suggested that optimal predefined pause duration between the dilute and neat gadolinium injection is 25 seconds or more if a contrast agent with high gadolinium concentration (1 mol/L) is used. Cardiac output had a negative but only moderate correlation with time width of the dilute CA curve and the time width of the dilute CA curve was significantly longer in the resting state than during stress. These finding suggest that the predefined pause should be prolonged for the rest perfusion scan if there was any overlap of the curves at stress. Theoretically, using a lower concentration of gadolinium contrast agent (e.g. 0.5 mol/L), the bolus profile should be wider than in the case of higher concentration of gadolinium contrast agent (e.g. 1.0 mol/L) because the weight dependent volume of contrast agent given to the patient is larger (e.g. If the patient's body weight is 60 kg and dose of gadolinium contrast is 0.1 mmol/kg, 6 ml of Gd-DO3A-butrol is administered which is equivalent to 12 ml of Gd-DTPA). Therefore, a longer pause between the two injections should be used.

The current approach requires substantial "user" interaction. In the present study, there were 3 technical errors, in two cases the dilute gadolinium agent and the neat contrast agent syringes were confused. A repetition of these errors was avoided by carefully labelling each syringe. Another error was observed at a remote site and related to the manual contrast injection at a wrong time. This occurred during one of the first studies and might be explained by relative inexperience with the method. Undergoing some simple and brief training before applying our methods during clinical scanning should overcome such "user" related problems. This dual-bolus injection scheme requires 6.9 ± 1.5 minutes for the preparation of gadolinium contrast agent and the set-up for power injector and lines. However, the perfusion scan is only extended by the duration of the predefined pause (i.e. ~25s in each perfusion scan). In our institute, standard stress-rest myocardial perfusion CMR set-up with normal single bolus injection scheme requires 3.2 ± 1.8 minutes for the preparation of gadolinium contrast agent and the set-up for power injector and lines. The dual-bolus injection scheme needs just a few more minutes on top of the normal set-up

In patients with low LV function (EF < 30%), the dual-bolus curves tend to overlap due to low cardiac output. In these cases, a longer predefined pause is required. Further investigation is required to ascertain whether the poor bolus profile in these patients still provides diagnostic and accurate MBF quantification. In contrast, our dual-bolus scheme worked well in a patient with LV EF of > 80%.

Only adenosine was tested as a pharmacological stress agent in the present paper. Recently, the Food and Drug Administration approved regadenoson for stress testing in conjunction with myocardial perfusion imaging(15). Regadenoson, unlike adenosine, is a selective A2A agonist that is given as an intravenous bolus at a fixed dose and causes myocardial blood flow and heart rate to peak shortly after injection followed by a slow reduction in myocardial blood flow and a decreasing heart rate after approximately 2 minutes. Potentially, this dual-bolus scheme can be applied to regadenosone if the timing of bolus injection of contrast agent and regadenosone is optimized. Further studies will be required to show that this practical dual-bolus approach works for this new vasodilator stress agent.

### Limitation

We would like to acknowledge the main limitation of this study. Inevitably the use of a manual contrast injection necessitates the presence of a physician within the MR scanner room at the time of injection. However, the presence of a physician is reassuring for the patient particularly during the stress perfusion.

## Conclusion

We have devised a universal dual-bolus injection scheme, for use in a clinical setting, that is independent of sophisticated double-head power injector function. The universal dual-bolus injection scheme is a feasible technique to obtain a reasonable arterial input function curve to calculate absolute quantification of myocardial blood flow.

## Competing interests

EN received major grant support from Philips Healthcare and Bayer Schering Pharma. The other authors declare that they have no competing interests'.

## Authors' contributions

MI designed the study protocol, carried out the CMR studies, analyzed the data and drafted the manuscript. AS, GM, AC, SH, MP, NM, HS, DL, BS, KA and SP helped to perform the MR studies and to draft the manuscript. EN designed the study protocol and drafted the manuscript. All authors read and approved the final manuscript.
